# Mayo Adhesive Probability (MAP) score of non-donated kidney aids in predicting post-operative renal function following donor nephrectomy

**DOI:** 10.1186/s12894-020-00695-2

**Published:** 2020-08-17

**Authors:** Katherine J. Cockerill, Amanda E. Kahn, Stacy M. Young, Colleen T. Ball, Martin L. Mai, C. Burcin Taner, Dana K. Perry, David D. Thiel

**Affiliations:** 1 Department of Urology, Mayo Clinic, 4500 San Pablo Road, Jacksonville, FL 32224 USA; 2 Department of Health Sciences Research, Mayo Clinic, Jacksonville, FL USA; 3 Division of Biomedical Statistics and Informatics, Mayo Clinic, Jacksonville, FL USA; 4 Department of Transplantation, Mayo Clinic, Jacksonville, FL USA

**Keywords:** Donor nephrectomy, MAP score, Renal function

## Abstract

**Background:**

To examine the association of preoperative Mayo Adhesive Probability (MAP) scores in the donor (MAP_d_) and non-donor kidneys (MAP_nd_) with post-donation renal function.

**Methods:**

Three hundred thirty-one patients undergoing hand assisted laparoscopic donor nephrectomy (HALDN) were reviewed. MAP_d_ and MAP_nd_ were obtained. Estimated glomerular filtration rate (eGFR) was recorded preoperatively and at 1 day, 1 month, and 6 months postoperatively.

**Results:**

Two hundred females and 131 males were evaluated with median BMI 26.4 kg/m^2^ (range 17.1–39.6) and median age 45 years (range 19–78). MAP_d_ score was 0 for 231 patients (69.8%) and > 0 for 100 patients (30.2%). MAP_nd_ score was 0 for 234 patients (70.7%) and > 0 for 97 patients (29.3%). The median preoperative eGFR was 86.6 ml/min/1.73m^2^ (range 48.8–138.4). After adjusting for preoperative eGFR, BMI, ASA score, and kidney sidedness, postoperative eGFR was associated with MAP score in the non-donated kidney (*p* = 0.014) but not in the donated kidney (*p* = 0.24). Compared to donors with MAP_nd_ = 0, donors with a MAP_nd_ > 0, mean eGFR was − 2.33 ml/min/1.73m^2^ lower at postoperative day 1 (95% CI − 4.24 to − 0.41, *p* = 0.018), − 3.02 ml/min/1.73m^2^ lower at 1 month (95% CI − 5.11 to − 0.93, *p* = 0.005), and − 2.63 ml/min/1.73m^2^ lower at 6 months postoperatively (95% CI − 5.01 to − 0.26, *p* = 0.030).

**Conclusions:**

MAP score > 0 in the non-donated kidney is associated with worse renal function in the 6 months following HALDN.

## Background

Recent studies have evaluated the impact of various preoperative patient-specific and imaging-derived characteristics on postoperative renal function following donor nephrectomy. Examinations of large cohorts of patients suggest that age, male sex, and body mass index (BMI) are predictive of decreased postoperative renal function in patients undergoing donor nephrectomy [1–4]. However, other studies have shown that BMI has no effect on long term glomerular filtration rate (eGFR) in the donor nephrectomy population [5–8]. Lee et al. examined the effect of imaging-specific characteristics on postoperative glomerular filtration rate and demonstrated a correlation between postoperative recovery of renal function and visceral/subcutaneous adipose tissue measurements [9].

Adherent perinephric fat (APF) has been associated with surgical complexity and longer operative times in patients undergoing partial nephrectomy [10]. The Mayo Adhesive Probability (MAP) score was originally designed as a simple, accurate, image-based scoring system to predict the presence of troublesome APF during partial nephrectomy [11, 12]. Higher MAP score has previously been shown to be associated with longer operative time in patients undergoing hand-assisted laparoscopic donor nephrectomy [13]. The MAP score is calculated based on image-derived measures of perinephric fat distance and severity of perinephric stranding to achieve a MAP score of 0–5 [11]. Narita et al. outlined risk factors for APF in healthy patients undergoing laparoscopic donor nephrectomy. In their study, MAP score components (perinephric fat area and stranding) were correlated with the presence of APF during donor nephrectomy [14]. Motivated by these findings, we examined the relationship between MAP score and post-donation renal function in healthy patients undergoing hand-assisted laparoscopic donor nephrectomy (HALDN).

## Methods

### Hand-assisted laparoscopic donor nephrectomy

Hand-assisted laparoscopic donor nephrectomy (HALDN) was performed as previously described by fellowship-trained transplant surgeons at one institution using a hand-port and two additional trocars [13, 15]. The perinephric fat was dissected and the renal hilum exposed in each case. The ureter was controlled distally with a clip and sharply divided. An endovascular stapler was used to take the renal artery and the renal vein separately.

### Data collection

The data set is similar to that used in our previous manuscript evaluating the relationship between MAP score and operative time in HALDN [13]. Following approval by the Mayo Clinic Institutional Review Board, 331 consecutive patients who underwent HALDN between January 2007 and April 2017 were evaluated. Data collected include patient characteristics (age, sex, race, MAP scores for donated [MAP_d_] and non-donated kidneys [MAP_nd_]), preoperative laboratory values (estimated glomerular filtration rate [eGFR], creatinine, hemoglobin [Hgb]), American Society of Anesthesiologists [ASA] score, sidedness of donor kidney, operative time), and measurement of postoperative renal function (eGFR and creatinine at postoperative day 1 [POD1], 1 month, and 6 months, calculated according to the 2006 MDRD study equation) [16]. Patients with preoperative eGFR< 60 mL/min/1.73m^2^ were still considered candidates for donor nephrectomy at our institution based on measured iothalamate or creatinine clearance greater than or equal to the lower 5th percentile when age-adjusted.

### Calculation of MAP score

MAP score was calculated as described in our previous manuscripts evaluating MAP score and its relationship to adherent perinephric fat, renal cell carcinoma progression free survival, and HALDN [11, 13, 17]. An independent reviewer evaluated the preoperative imaging (CT or T1-weighted MRI) for each patient undergoing HALDN to calculate MAP scores. The MAP_d_ score was calculated for the patient’s donated kidney and the MAP_nd_ score was calculated for the patient’s non-donated kidney. MAP scores were calculated for every patient utilizing the measurement of posterior renal fat thickness and the measure of severity of perinephric stranding. Perinephric fat thickness was measured at the level of the renal vein for each kidney. Posterior renal fat was measured at this level as a direct line from the level of the renal capsule to the posterior abdominal wall [11]. This variable was measured in centimeters (< 1 cm = 0 points, 1.1–1.9 cm = 1 point, > 2.0 cm = 2 points).

Perinephric stranding was identified as soft tissue attenuation in the fat surrounding the kidney. This was graded according to severity if present (0 = no stranding, 2 = thin mild stranding, 3 = diffuse stranding). The MAP score was calculated in each scenario as the sum of the values obtained for posterior renal fat and perinephric stranding to obtain a score 0–5 [11].

### Statistical analysis

Box plots were constructed to show the distribution of preoperative eGFR according to MAP score. For evaluating associations of MAP score with renal function, MAP_d_ and MAP_nd_ scores were categorized as either 0 or > 0 given the low number of patients with MAP scores of 1–5. All analyses were done separately for MAP_d_ and MAP_nd_. For cross-sectional associations of MAP score with preoperative eGFR we used linear regression. We used mixed effects regression models with preoperative eGFR as a covariate to evaluate associations of MAP score with postoperative eGFR. Mixed effects models additionally included random patient effects for intercepts and slopes, two indicator variables to represent three postoperative time points, three indicator variables to represent MAP score (> 0 vs. 0) at each postoperative time point. All regression-based models were adjusted for body mass index, ASA score, and sidedness of the donated kidney. Supplementary Table 1 shows associations of MAP score with postoperative eGFR adjusting for one covariate at a time. Our final model included all pre-specified covariates with the exception of age, sex, and race as these are used to calculate eGFR. Likelihood ratio tests (LRT) with 3 degrees of freedom were used to evaluate the overall association of MAP score on postoperative renal function. Confidence intervals and *p*-values corresponding to the difference in means and difference in slopes were two-sided and based on the t-distribution. *P*-values less than 0.05 were considered statistically significant. Fitted values obtained from regression models refer to a hypothetical “average” donor where baseline covariates are equal to the mean value of all patients included in the model. SAS statistical software (version 9.4, SAS Institute Inc., Cary, NC) was used for all statistical analyses.

## Results

A total of 331 patients who underwent HALDN were included in the analysis. Median age was 45 years (range 19 to 78 years) and BMI was 26.4 kg/m^2^ (range 17.1 to 39.6 kg/m^2^). A large majority of the patients underwent left-sided donor nephrectomy (89.4%). Two hundred patients were female (60.4%). Of note, patient comorbidities were minimal given the rigorous screening process at our institution prior to consideration of donor nephrectomy. Two hundred and thirty one patients had a MAP_d_ score of 0 (69.8%) and 100 patients had a MAP_d_ score of 1–5 (30.2%). Two hundred and thirty four patients had a MAP_nd_ of 0 (70.7%) and 97 patients had MAP_nd_ of 1–5 (29.3%). Table 1 summarizes additional preoperative patient characteristics, ASA scores, and MAP_d_ and MAP_nd_ scores of the 331 renal donors.

**Table 1:** Preoperative characteristics of the full cohort of patients who had a donor nephrectomy according to Mayo Adhesive Probability Score

Baseline characteristic	MAP Score 0 (*N* = 209)	MAP Score 1–5 (*N* = 122)
Age (years)	42.3 ± 12.4	48.6 ± 9.1
Sex		
Female	159 (76.1%)	41 (33.6%)
Male	50 (23.9%)	81 (66.4%)
Black race		
No	176 (84.2%)	110 (90.2%)
Yes	33 (15.8%)	12 (9.8%)
Body mass index (kg/m^2^)	25.6 ± 3.9	28.3 ± 3.9
ASA score		
1	135 (64.6%)	70 (57.4%)
2	72 (34.4%)	47 (38.5%)
3	2 (1.0%)	5 (4.1%)
Donated kidney		
Right	23 (11.0%)	12 (9.8%)
Left	186 (89.0%)	110 (90.2%)
eGFR, ml/min/1.73m^2^	88.9 ± 16.8	82.7 ± 14.3

Abbreviations: *MAP* Mayo Adhesive Probability, *ASA* American Society of Anesthesiologists, *eGFR* estimated glomerular filtration rate. The sample mean ± SD is shown for continuous variables. Number (percentage of patients) is shown for categorical variables

**Fig. 1:**
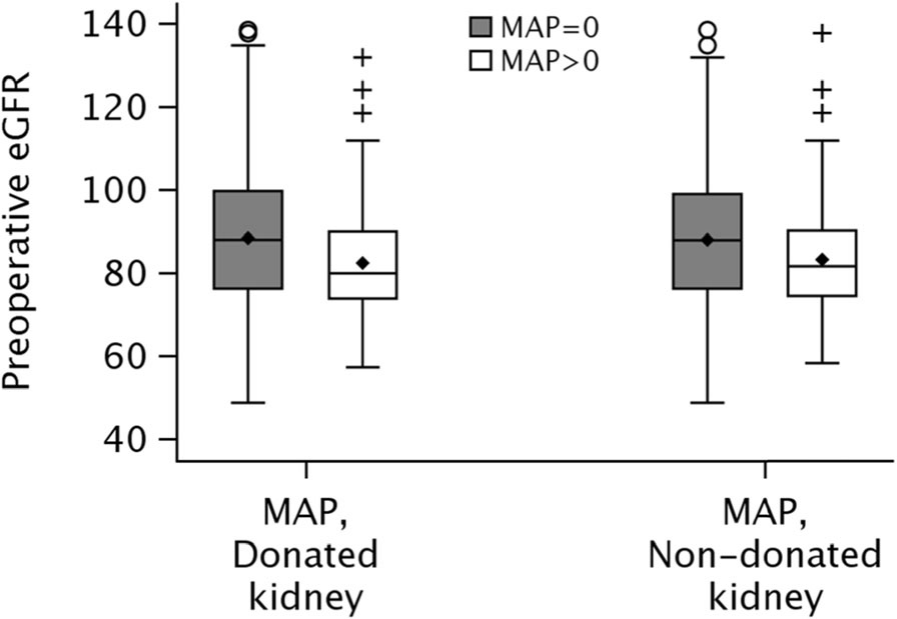
Box plots of preoperative estimated glomerular filtration rate (eGFR) according to Mayo Adhesive Probability (MAP) score in the donated kidney and non-donated kidney. Units of measurement for eGFR are ml/min/1.73m^2^

Among the entire cohort, the preoperative eGFR ranged from 48.8 to 138.4 ml/min/1.73m^2^ (mean = 86.6 ml/min/1.73m^2^, *n* = 331). The distribution of preoperative eGFR is presented in Fig. 1 according to MAP score (0 or > 0) and kidney type (MAP_d_ or MAP_nd_). Associations of MAP score with preoperative and postoperative renal function with adjustment for preoperative covariates are shown in Table 2. Donors with a MAP_d_ > 0 had a lower mean eGFR by 5.87 ml/min/1.73m^2^ lower (95% CI 2.02 to 9.71 ml/min/1.73m^2^) compared to kidney donors with a MAP_d_ = 0 in the donated kidney. When comparing eGFR according to MAP_nd_ score in the non-donated kidney, mean eGFR was 4.59 ml/min/1.73m^2^ lower (95% CI 0.63 to 8.54 ml/min/1.73m^2^) in those with a MAP_nd_ > 0 compared to those with MAP_nd_ = 0.

**Table 2:** Association of Mayo Adhesive Probability Score with Renal Function Before and After Donor Nephrectomy

Timing of eGFR measurement	MAP_**d**_ (> 0 vs. 0)		MAP_**nd**_ (> 0 vs. 0)	
	Difference in mean eGFR (95% CI), ml/min/1.73m^2^	P	Difference in mean eGFR (95% CI), ml/min/1.73m^2^	P
Preoperative^a^	−5.87 (−9.71 to −2.02)	0.003	−4.59 (−8.54 to −0.63)	0.023
Postoperative^b^		0.24		0.014
Day 1^c^	−0.44 (−2.33 to 1.46)	0.65	−2.33 (−4.24 to − 0.41)	0.018
1 Month ^c^	−1.68 (−3.80 to 0.45)	0.12	−3.02 (−5.11 to − 0.93)	0.005
6 Month ^c^	−1.99 (− 4.31 to 0.33)	0.092	−2.63 (− 5.01 to − 0.26)	0.030

*MAP*_*d*_ Mayo Adhesive Probability score in the donated kidney, *MAP*_*nd*_ Mayo Adhesive Probability score in the non-donated kidney, *eGFR* estimated glomerular filtration rate, *CI* confidence interval

^a^ Associations with preoperative renal function were estimated from linear regression models with adjustment for body mass index, ASA score, and sidedness of the donated kidney

^b^ The overall association of MAP score with postoperative renal function was evaluated using a likelihood ratio test with 3 degrees of freedom comparing the full mixed effects regression model to a model without the 3 MAP terms (one for each postoperative time point)

^c^ Associations with renal function at each postoperative time point were estimated from mixed effects regression models with adjustment for preoperative eGFR, body mass index, ASA score, and sidedness of the donated kidney

We also investigated the small cohort of patients (*n* = 10) with a high grade MAP_nd_ score (3 to 5). This cohort had a median preoperative eGFR of 75.3 ml/min/1.73m^2^ (range, 62.2–87.3 ml/min/1.73m^2^) with a median preoperative creatinine of 1.0 mg/dL (range, 0.9–1.2 mg/dL). At 1 month postoperatively, the median eGFR was 41.6 ml/min/1.73m^2^ (range 35.9–57.1 ml/min/1.73m^2^, *n* = 9) with a creatinine of 1.7 mg/dL (range 1.3–1.9 mg/dL, *n* = 9). At 6 months postoperatively, the median eGFR was 42.9 ml/min/1.73m^2^ (range 38.9–58.4 ml/min/1.73m^2^, *n* = 7) with a median creatinine of 1.6 mg/dL (range 1.3–1.7 mg/dL). No patient from this high risk group had an eGFR greater than 60 ml/min/1.73m^2^ at 1 month or 6 months postoperatively.

**Fig. 2:**
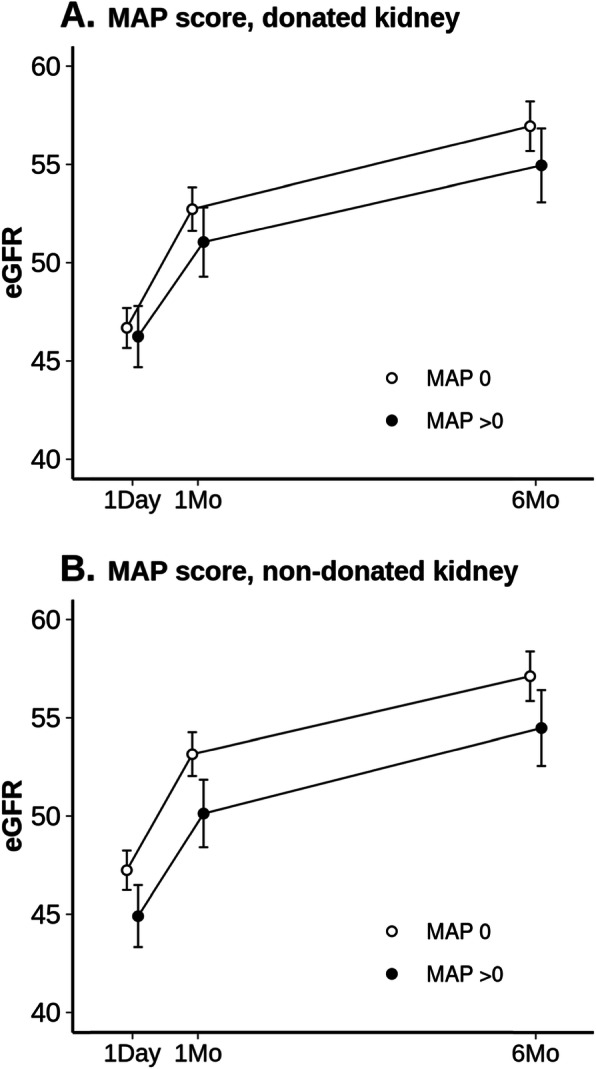
Estimated glomerular filtration rate (eGFR) after hand assisted laparoscopic donor nephrectomy according to Mayo Adhesive Probability (MAP) score in the donated kidney (Panel **a**) and non-donated kidney (Panel **b**). Fitted values of eGFR, measured in units of ml/min/1.73m^2^, were obtained from mixed effects regression models. These fitted values refer to a hypothetical average patient where covariates (preoperative eGFR, body mass index, ASA score, and kidney sidedness) are set equal to their mean values across the entire cohort). Vertical bars represent the 95% confidence intervals

We used mixed effects linear regression models to evaluate the impact of MAP score on postoperative eGFR while adjusting for preoperative eGFR and baseline covariates as displayed in Table 2. The overall association of MAP score with postoperative eGFR was statistically significant in the non-donated kidney (LRT *p* = 0.014, df = 3) but not in the donated kidney (LRT *p* value = 0.24). Compared to those with a MAP_nd_ = 0, donors with a MAP_nd_ > 0 had evidence of significantly lower eGFR at POD1 (difference in means: − 2.33 ml/min/1.73m^2^; 95% CI − 4.24 to − 0.41 ml/min/1.73m^2^; *p* = 0.018), 1 month follow-up (difference in means: − 3.02 ml/min/1.73m^2^; 95% CI − 5.11 to − 0.93 ml/min/1.73m^2^; *p* = 0.005), and 6 months follow-up (difference in means: − 2.63 ml/min/1.73m^2^; 95% CI − 5.01 to − 0.26 ml/min/1.73m^2^; *p* = 0.030). We did not find evidence of an association of MAP_nd_ with the change in eGFR from POD1 (slopes) to either 1 month (difference in slopes [MAP_nd_ > 0 vs. MAP_nd_ = 0]: − 0.67 ml/min/1.73m^2^; 95% CI − 2.86 to 0.77 ml/min/1.73m^2^; *p* = 0.53) or 6 month follow-up (difference in slopes [MAP_nd_ > 0 vs. MAP_nd_ = 0]: − 0.31 ml/min/1.73m^2^; 95% CI − 2.70 to 2.08 ml/min/1.73m^2^; *p* = 0.80). Figure 2 displays the fitted estimates of postoperative eGFR according to MAP score in the donated kidney (panel A) and MAP score in the non-donated kidney (panel B). In a separate subgroup analysis, we obtained fitted estimates of postoperative eGFR according to MAP score separately for males and females (Supplementary Figure 1).

The MDRD 2006 formula, by definition, inherently demonstrates a correlation between age, sex, and race with eGFR [15]. Six months following nephrectomy, 47% (47/100) of donors younger than 45 years, 80% (79/99) of donors 45 to 65 years, and 100% (9/9) of donors over 65 years of age presented with an eGFR less than 60 mL/min/1.73m^2^.

## Discussion

In preoperative examination of candidates for donor nephrectomy, assessment of patient-specific factors is crucial in preparation for surgery [18]. Part of that workup includes an analysis of the patient’s BMI. There have been conflicting studies regarding the association between donor BMI and postoperative renal compromise [1–8]. Motivated by these conflicting studies, we examine the association of kidney MAP score with postoperative renal function in healthy donors.

The MAP score serves as a patient-specific and donor site-specific score that is widely applicable to the kidney donor, given that most renal donors have pre-operative cross-sectional imaging (CT scan or MRI). It has also been previously discovered to be an accurate imaged-based predictor of intraoperative APF and postoperative outcomes in patients undergoing laparoscopic and open partial nephrectomy [11, 12, 18]. Narita et al. found a correlation between MAP score variables and the identification of APF at the time of living-donor nephrectomy in their evaluation of 92 patients [14]. Lee et al. examined the correlation between CT measurement of visceral adipose tissue and postoperative renal function in their study of 75 living donors. Their study found a correlation between visceral to subcutaneous adipose tissue measurements and postoperative recovery of eGFR [9]. Studies have shown that BMI is not always a reliable measure of adipose tissue composition, especially when the visceral fat is in question [17, 19, 20]. Additionally, studies have shown that visceral fat is the most metabolically active type of fat [21, 22]. We believe the surgery specific measurement of perinephric fat and perinephric stranding may have more of an association with postoperative renal function than BMI alone due to these being inherently kidney-specific measures. Due to its proximity to the kidney and metabolically active nature, we believe an analysis of visceral fat by the MAP score may be a more effective measure than BMI when trying to understand how body fat composition affects kidney function. Results of our study indicate that MAP score in the non-donated kidney contributes to the prediction of postoperative renal function beyond the association of BMI with postoperative renal function (Likelihood ratio test *p* = 0.014, comparing the full model to the model without MAP score, Table 2). In addition, perinephric stranding can be indicative of underlying inflammatory or metabolic processes that can also ultimately affect intraoperative dissection and renal function. This may be most relevant to renal function on POD 1 given intraoperative challenges presented by adherent perinephric fat. However, our study also incorporates these concepts in the examination of long term renal function in a cohort of patients undergoing HALDN.

Additionally, age and male sex have previously been correlated with decreased postoperative donor renal function [3, 23]. By definition, the MDRD 2006 equation correlates age, sex, and race with eGFR because these variables are included in the calculation of eGFR.

Our study findings show that patients with MAP_nd_ scores from 1 to 5 were significantly associated with worse post-donation renal function than patients with MAP_nd_ scores of 0 (*p* = 0.014). After donor nephrectomy, recovery of renal function is dependent upon adaptive hypertrophy and hyperfiltration of the remaining kidney. This may explain why high MAP_nd_ scores proved to be significantly associated with diminished renal function following HALDN. The MAP score enables evaluation of the thickness and quality of the fat surrounding the non-donated kidney. An evaluation by Yoon et al. demonstrated that an increased area of visceral fat is associated with decreased eGFR and renal function [24]. The use of the MAP_nd_ score quantifies the posterior perinephric fat, a quantifiable measure of visceral fat, that may be more specific in predicting remaining renal function in kidney donors compared to BMI. We believe the MAP score may be utilized as an imaged-based adjunct to aid in the evaluation of potential donor renal dysfunction; however larger studies are needed for confirmation.

We believe the results from this study may contribute to how patients are evaluated for kidney donation in the future. The information may also contribute to patient counseling. For example, a healthy 58-year-old white male included in this study had a MAP_d_ of 4 and MAP_nd_ of 3. His preoperative eGFR was 76.7 ml/min/1.73m^2^ with a creatinine of 1 mg/dL. Postoperatively, his eGFR dropped to 41.6 ml/min/1.73m^2^ at 1 month, 41.5 ml/min/1.73m^2^ at 6 months, and 32.5 ml/min/1.73m^2^ at 12 months. All postoperative creatinine measurements were greater than or equal to 1.7. Due to his age, this patient will likely be living with chronic renal insufficiency for many years.

When evaluating patients who presented with a high grade MAP_nd_ score (3 to 5) (*n* = 12), every patient had a preoperative eGFR greater than 60 ml/min. Table 2 did reveal that patients with MAP of 1–5 also had a lower mean preoperative eGFR compared to patients with MAP = 0 (adjusted difference in means: − 4.59 ml/min/1.73m^2^). However, every patient with a high MAP score had a eGFR less than 60 ml/min postoperatively at 1 month or 6 months. Data available from 8 patients showed no improvement at 12 months postoperatively; no patients had an eGFR greater than 60 ml/min and none improved to within 10% of their preoperative eGFR baseline.

One limitation of our study is the low number of patients with high MAP scores. This is expected given our otherwise healthy population of kidney donors who are screened for potential diseases or malignancies that could contribute to a higher MAP score. The low number of patients with high MAP scores in this cohort precludes us from evaluating the impact of high MAP scores on postoperative renal function. However, the screening process that limits patients with a high MAP score also removes the potential confounding contribution of additional disease processes such as diabetes and hypertension on overall renal function. We did not have statistics within our dataset regarding smoking status, percentage of patients with diabetes, and other comorbidities that can affect long term renal function. This serves as a limitation to our study. Another limitation of our study is the limited number of females with a MAP score > 0. Previous studies have shown that men tend to have a greater amount of perirenal fat compared to women [25]. In a subgroup analysis we observed a similar pattern in the fitted values of postoperative eGFR in males and females, however the wide confidence intervals among females with a MAP score indicate a high level of uncertainty in those estimates (Supplementary Figure 1). Additionally, we recognize the limitation of the MDRD 2006 formula to most accurately calculate eGFR. This study was retrospective in nature therefore, only measurements considered “standard of care” were recorded and made available for analysis.

## Conclusion

The MAP scoring system can be universally applied to preoperative imaging in the evaluation of patients undergoing HALDN. Our study suggests that MAP scores greater than 0 in the non-donated kidney aid in estimating postoperative renal function. It is important to note that none of the 10 patients with a MAP score > 2 recovered baseline renal function or had post-donation eGFR > 60 ml/min/1.73m^2^.

## Supplementary information

**Additional file 1: Supplementary Figure 1.** Estimated glomerular filtration rate (eGFR) after hand assisted laparoscopic donor nephrectomy according to patient sex and Mayo Adhesive Probability (MAP) score in the donated kidney and non-donated kidney. Fitted values of eGFR, measured in units of ml/min/1.73m^2^, were obtained from mixed effects regression models separately for males and females. These fitted values refer to a hypothetical average patient where covariates (preoperative eGFR, body mass index, ASA score, and kidney sidedness) are set equal to their mean values, separately for males and females). Vertical bars represent the 95% confidence intervals.

**Additional file 2: Supplementary Table 1.** Exploration of the impact of preoperative characteristics on the association of Mayo Adhesive Probability score with postoperative renal function.

## Data Availability

The datasets used and/or analysed during the current study are available from the corresponding author on reasonable request.

## Abbreviations

eGFR: Estimated glomerular filtration rate
APF: Adherent perinephric fat
MAP: Mayo Adhesive Probability score
HALDN: Hand-assisted laparoscopic donor nephrectomy
MAP_d_: MAP scores for donated
MAP_nd_: Non-donated kidneys
BMI: Body mass index
Hgb: Hemoglobin
WBC: White blood cell count
ASA: American Society of Anesthesiologists score
POD1: Postoperative day 1
