# Supplementary material for: Mayo Adhesive Probability (MAP) score of non-donated kidney aids in predicting post-operative renal function following donor nephrectomy
Source: BMC Urol. 2020 Aug 17;20:124. doi: 10.1186/s12894-020-00695-2 (PMC7433049; doi:10.1186/s12894-020-00695-2)

**Supplementary Figure 1. Estimated glomerular filtration rate (eGFR) after hand assisted laparoscopic donor nephrectomy according to patient sex and Mayo Adhesive Probability (MAP) score in the donated kidney and non-donated kidney.** *Fitted values of eGFR, measured in units of ml/min/1.73m^2^, were obtained from mixed effects regression models separately for males and females. These fitted values refer to a hypothetical average patient where covariates (preoperative eGFR, body mass index, ASA score, and kidney sidedness) are set equal to their mean values, separately for males and females).Vertical bars represent the 95% confidence intervals.*


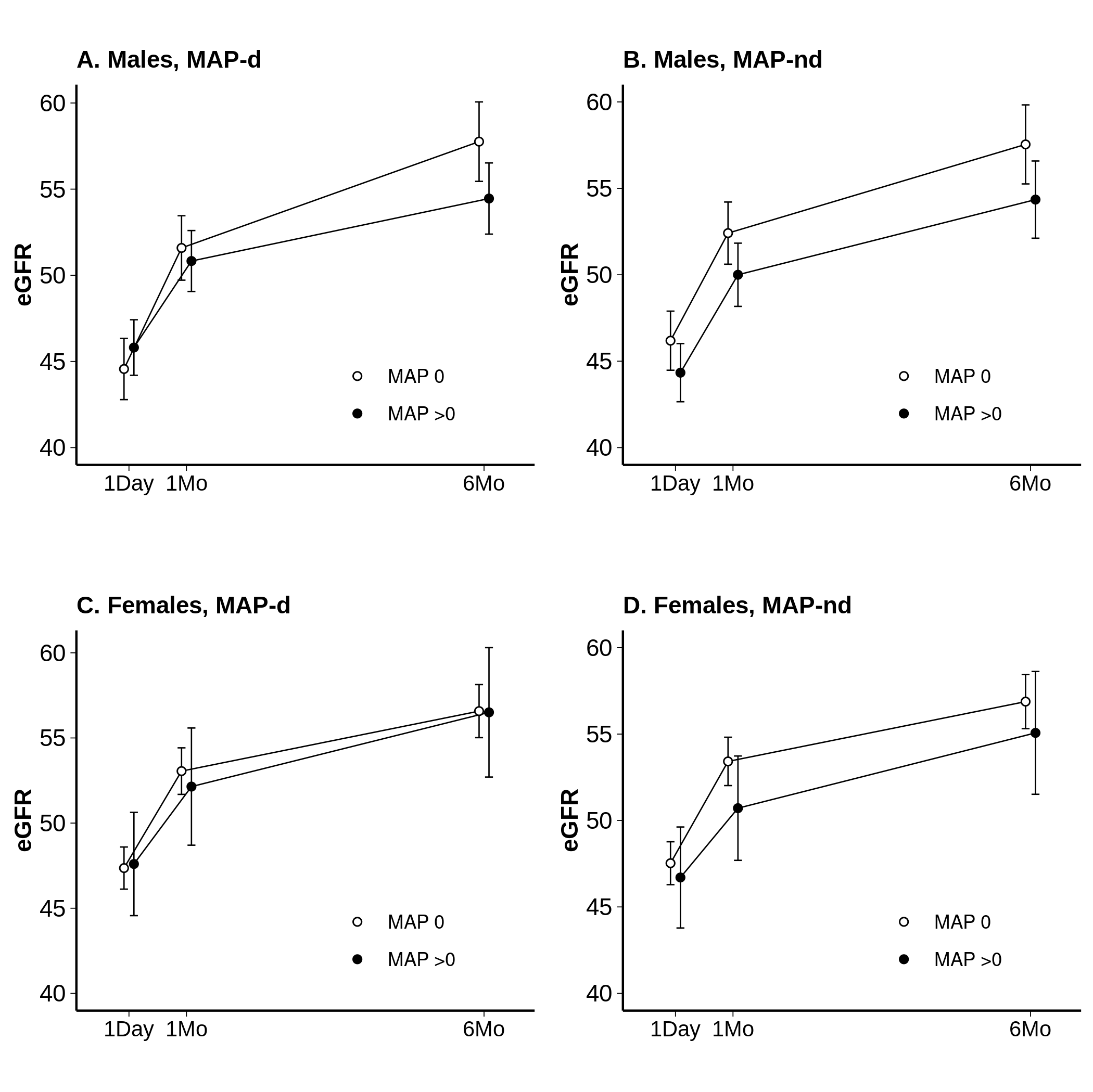

Supplement: Supplementary file 1 — Additional file 1: Supplementary Figure 1. Estimated glomerular filtration rate (eGFR) after hand assisted laparoscopic donor nephrectomy according to patient sex and Mayo Adhesive Probability (MAP) score in the donated kidney and non-donated kidney. Fitted values of eGFR, measured in units of ml/min/1.73m2, were obtained from mixed effects regression models separately for males and females. These fitted values refer to a hypothetical average patient where covariates (preoperative eGFR, body mass index, ASA score, and kidney sidedness) are set equal to their mean values, separately for males and females). Vertical bars represent the 95% confidence intervals. [file 12894_2020_695_MOESM1_ESM.docx]
