# Supplementary material for: Mayo Adhesive Probability (MAP) score of non-donated kidney aids in predicting post-operative renal function following donor nephrectomy
Source: BMC Urol. 2020 Aug 17;20:124. doi: 10.1186/s12894-020-00695-2 (PMC7433049; doi:10.1186/s12894-020-00695-2)
Supplement: Supplementary file 2 — Additional file 2: Supplementary Table 1. Exploration of the impact of preoperative characteristics on the association of Mayo Adhesive Probability score with postoperative renal function. [file 12894_2020_695_MOESM2_ESM.docx]

**Supplementary Table 1. Exploration of the impact of preoperative characteristics on the association of Mayo Adhesive Probability score with postoperative renal function.**

|  | Association of MAP Score (>0 vs. 0) with Postoperative eGFR | | | | | |
| --- | --- | --- | --- | --- | --- | --- |
|  | Day 1 | | 1 Month | | 6 Month | |
|  | Difference (95% CI), ml/min/1.73m^2^ | P | Difference (95% CI), ml/min/1.73m^2^ | P | Difference (95% CI), ml/min/1.73m^2^ | P |
| **MAP score (>0 vs. 0), Donated Kidney** |  |  |  |  |  |  |
| Adjusted for preoperative eGFR | -0.48 (-2.30 to 1.35) | 0.61 | -2.10 (-4.13 to -0.07) | 0.043 | -2.42 (-4.66 to -0.18) | 0.034 |
| Adjusted for preoperative eGFR plus: |  |  |  |  |  |  |
| Age | -0.08 (-1.94 to 1.78) | 0.93 | -1.15 (-3.22 to 0.91) | 0.27 | -1.07 (-3.24 to 1.11) | 0.33 |
| Sex | 0.87 (-1.12 to 2.85) | 0.39 | -1.32 (-3.53 to 0.88) | 0.24 | -2.33 (-4.81 to 0.14) | 0.065 |
| Race | -0.46 (-2.28 to 1.36) | 0.62 | -2.05 (-4.07 to -0.02) | 0.047 | -2.20 (-4.44 to 0.03) | 0.054 |
| BMI | -0.40 (-2.30 to 1.50) | 0.68 | -1.78 (-3.90 to 0.35) | 0.10 | -1.93 (-4.25 to 0.40) | 0.10 |
| ASA score | -0.53 (-2.35 to 1.29) | 0.57 | -1.96 (-3.99 to 0.08) | 0.059 | -2.49 (-4.74 to -0.24) | 0.030 |
| Kidney sidedness | -0.47 (-2.30 to 1.35) | 0.61 | -2.10 (-4.13 to -0.07) | 0.043 | -2.44 (-4.67 to -0.21) | 0.032 |
| BMI, ASA score, and Kidney sidedness^a^ | -0.44 (-2.33 to 1.46) | 0.65 | -1.68 (-3.80 to 0.45) | 0.12 | -1.99 (-4.31 to 0.33) | 0.092 |
|  |  |  |  |  |  |  |
| **MAP score (>0 vs. 0), Non-Donated Kidney** |  |  |  |  |  |  |
| Adjusted for preoperative eGFR | -2.15 (-3.97 to -0.34) | 0.020 | -3.34 (-5.34 to -1.34) | 0.001 | -3.16 (-5.41 to -0.92) | 0.006 |
| Adjusted for preoperative eGFR plus: |  |  |  |  |  |  |
| Age | -1.88 (-3.72 to -0.04) | 0.045 | -2.57 (-4.58 to -0.55) | 0.013 | -2.19 (-4.34 to -0.04) | 0.046 |
| Sex | -1.28 (-3.22 to 0.66) | 0.19 | -2.82 (-4.92 to -0.71) | 0.009 | -3.13 (-5.54 to -0.71) | 0.011 |
| Race | -2.13 (-3.94 to -0.32) | 0.021 | -3.27 (-5.27 to -1.28) | 0.002 | -2.94 (-5.19 to -0.70) | 0.011 |
| BMI | -2.27 (-4.19 to -0.35) | 0.021 | -3.15 (-5.24 to -1.06) | 0.003 | -2.67 (-5.05 to -0.28) | 0.029 |
| ASA score | -2.23 (-4.05 to -0.42) | 0.016 | -3.18 (-5.19 to -1.18) | 0.002 | -3.21 (-5.46 to -0.96) | 0.006 |
| Kidney sidedness | -2.16 (-3.97 to -0.34) | 0.02 | -3.35 (-5.34 to -1.35) | 0.001 | -3.14 (-5.37 to -0.90) | 0.006 |
| BMI, ASA score, and Kidney sidedness^a^ | -2.33 (-4.24 to -0.41) | 0.018 | -3.02 (-5.11 to -0.93) | 0.005 | -2.63 (-5.01 to -0.26) | 0.030 |
| MAP, Mayo adhesive probability; eGFR, estimated glomerular filtration rate; The differences in mean eGFR (MAP>0 minus MAP=0) were estimated from mixed effects regression models with random patient intercepts and slopes.  ^a^ The final model did not include age, sex, or race since these are used to calculate eGFR. | | | | | | |
